# Correlates of compliance with recommended levels of physical activity in children

**DOI:** 10.1038/s41598-017-16525-9

**Published:** 2017-11-28

**Authors:** Thayse Natacha Gomes, Peter T. Katzmarzyk, Donald Hedeker, Mikael Fogelholm, Martyn Standage, Vincent Onywera, Estelle V. Lambert, Mark S. Tremblay, Jean-Philippe Chaput, Catrine Tudor-Locke, Olga Sarmiento, Victor Matsudo, Anura Kurpad, Rebecca Kuriyan, Pei Zhao, Gang Hu, Timothy Olds, Carol Maher, José Maia

**Affiliations:** 1 CIFI2D, Faculty of Sport, University of Porto, Porto, Portugal; 2 Pennington Biomedical Research Center, Baton Rouge, Louisiana United States; 3 Department of Public Health Sciences, University of Chicago, Chicago, Illinois United States; 4 Department of Food and Environmental Sciences, University of Helsinki, Helsinki, Finland; 5 University of Bath, Bath, United Kingdom; 6 Department of Recreation Management and Exercise Science, Kenyatta University, Nairobi, Kenya; 7 UCT/MRC Research Unit for Exercise Science and Sports Medicine, Faculty of Health Sciences, University of Cape Town, Cape Town, South Africa; 8 Healthy Active Living and Obesity Research Group, Children’s Hospital of Eastern Ontario Research Institute, Ottawa, Ontario, Canada; 9 Department of Kinesiology, School of Public Health and Health Sciences, University of Massachusetts Amherst, Amherst, Massachusetts United States; 10 Universidad de los Andes, Bogota, Colombia; 11 Centro de Estudos do Laboratório de Aptidão Física de São Caetano do Sul (CELAFISCS), São Paulo, Brazil; 12 St. Johns Research Institute, Bangalore, India; 13 Tianjin Women’s and Children’s Health Center, Tianjin, China; 14 Alliance for Research in Exercise, Nutrition and Activity (ARENA)/Sansom Institute, University of South Australia, Adelaide, Australia

## Abstract

The purpose of this study was to describe children’s daily compliance with moderate-to-vigorous physical activity (MVPA) recommendations across a week in different parts of the world, and to identify individual- and school-level correlates that may explain differences in daily MVPA compliance. The sample included 6553 children aged 9–11 years from 12 countries, and multilevel statistical analyses were used, including both child- and school-level variables. Most children did not comply with the MVPA guidelines on a daily basis: Chinese children complied the least, whereas Finnish, Australian, Colombian, UK, and Kenyan children complied the most. Boys (rate ratio [RR] = 1.47) and children with higher unhealthy diet scores (RR = 1.08) complied more, but overweight/obese children (RR = 0.81), earlier maturing children (RR = 0.93), and those who spent more time in screen activities (RR = 0.98) and sleeping (RR = 0.96) had the lowest compliance. At the school level, children with access to playground or sport equipment (RR = 0.88, for both) tended to comply less, whereas those with access to a gymnasium outside the school hours complied more with the MVPA guidelines (RR = 1.14). Significant between-country differences in children’s daily MVPA compliance were observed, reflecting not only site characteristics, but also the importance of individual traits and local school contexts.

## Introduction

The current global physical activity guidelines from the World Health Organization (WHO) recommend that children and youth accumulate at least 60 minutes of moderate-to-vigorous physical activity (MVPA) daily in order to achieve health benefits^1^. However, questionnaire-based data show that 80.3% of adolescents worldwide are not complying with these recommendations^2^.

Multi-country data capturing children’s physical activity and sedentariness variability have produced inconsistent conclusions^3–5^. For example, in a study of European youth from five countries (Belgium, Greece, Hungary, the Netherlands, and Switzerland), Greek children from both sexes spent, in general, more time in sedentary behaviours, while Swiss children not only spent more time in MVPA but also reported the highest compliance with MVPA guidelines (12.5% in girls, 27.8% in boys)^3^. Further, Riddoch *et al*.^4^ and Cooper *et al*.^5^ have both reported that, on average, Norwegian children were the most active (when compared with Danish, Estonian, and Portuguese youth^4^, or when compared with Australian, Danish, English, Estonian, Portuguese, and US youth^5^) and, in general, complied more with MVPA guidelines^5^. Overall, the prevalence of youth, aged 5–17 years, meeting MVPA guidelines on a daily basis ranged from 1.9% in girls to 9% in boys when using every monitored and valid day^5^. However, these data came from different sources where accelerometer monitoring ranged from 2 to 7 days; further, the exact number of days children complied with the guidelines was not explicitly reported. These worldwide differences in children’s physical activity levels can be traced to heterogeneity in their biological, behavioural and environmental characteristics. It has been consistently reported that sex^6^, weight status^6^, biological maturation^7^, sleep time^8^, and nutritional habits^9^ are relevant correlates of MVPA. Further, children spend much of their waking time at school. It is expected that the school environment provides ample opportunities for children to be physically active because of its social and physical settings, namely campus size or playground areas, sports equipment or facilities, recess periods/lunch breaks, and promotion of students’ active transportation^10,11^. Further, dissimilarities across schools’ contexts, or environmental sites within and between countries, may play important roles in explaining differences in children’s MVPA levels^12^.

Notwithstanding the international recommendation for children and adolescents to be physically active, it is interesting to note from previous research, even in multi-centre studies, that little attention has been given to determining children’s compliance with MVPA guidelines on a daily basis, especially across a whole week. Rather, the focus has been mainly on differences, considering measured and valid days, in mean MVPA values, or total physical activity averages, across countries^3–5^. Measurement summaries of a whole/typical week may not be sensitive enough to provide information about children’s overall physical activity compliance rates. In addition, as previously reported in Portuguese children^13^, examining average MVPA does not provide any information about how many days a child complies with the recommendations, and information regarding day-to-day variability across a whole week could provide a better understanding of children’s MVPA patterns. Furthermore, since children’s MVPA levels differ across countries^4,5^, it is possible that differences in their correlates might be country-specific, since cross-cultural differences are expected in their behavioural habits, socio-demographic and geographical characteristics, meaning that children’s MVPA compliance rates (and not only MVPA patterns or levels) may differ between countries. Taken as a whole, these country-level differences reflect differences in daily life routines, and their correlates are also expected to be specific in their effect sizes. For example, in our earlier report using data from the International Study of Childhood Obesity, Lifestyle and the Environment (ISCOLE), country differences were observed in children’s use of active transportation^14^ and in average daily MVPA^15^. However, these reports also focused their attention on average values. The present study aims (1) to investigate children’s daily compliance with daily MVPA recommendations across a week in 12 different study sites with diverse human development/sociodemographic characteristics from all major world regions; and (2) to identify individual-level and school-level correlates across countries that may explain differences in daily MVPA compliance.

## Results

Descriptive statistics for level-1 and level-2 covariates are provided in Tables 1 and 2, respectively. In general (see Table 1), children were about 2 years from their estimated PHV; children from United Kingdom (UK) and Australian sites were closest to their PHV, while those from the Kenyan site were the furthest (−2.5 years to PHV). Regarding dietary patterns, children from the Colombia site showed the lowest healthy diet score, and those from the Canadian site reported the highest; children from the Finnish site had the lowest unhealthy diet scores, while those from the South Africa site showed the highest. Averaged across the week and participants, in seven of the country sites children exceeded the ≥60 min/day MVPA guideline; the lowest average daily MPVA was observed in children from the Chinese site, while those from Kenya and Finland sites showed the highest daily average. In addition, screen time and sleeping varied between about 2–4 and 8–9 hours per day across sites, respectively. The prevalence of overweight/obesity (Table 1) ranged from 19.9% (Kenyan site) to 46.6% (Portuguese site), and in 7 of the 12 country sites the prevalence was ≥30%.

**Table 1:** Descriptive statistics (means ± standard deviations or percentage 95%CI) of child-level variables by study site.

Country (site)	BMI (kg∙m^−2^)	Maturity Offset (years from PHV)	Healthy Diet score	Unhealthy Diet score	MVPA (min∙day^−1^)	Screen Time (hours∙day^−1^)	Sleep (hours∙day^−1^)	Percentage of Overweight/Obese	MVPA compliance days
Australia (Adelaide)	18.8 ± 3.4	−1.7 ± 0.9	0.2 ± 1.0	−0.3 ± 0.7	64.8 ± 23.2	2.8 ± 1.8	9.4 ± 0.7	37.3% (33.0–41.7)	3.3 ± 2.1
Brazil (São Paulo)	19.7 ± 4.4	−2.0 ± 1.0	−0.4 ± 1.0	0.1 ± 0.9	59.5 ± 26.1	3.7 ± 2.3	8.6 ± 0.8	44.9% (40.5–49.5)	2.9 ± 2.2
Canada (Ottawa)	18.3 ± 3.3	−1.9 ± 0.9	0.5 ± 1.0	−0.5 ± 0.6	58.7 ± 19.3	2.5 ± 1.9	9.1 ± 0.8	30.8% (26.9–34.9)	2.9 ± 2.1
China (Tianjin)	18.9 ± 4.2	−2.3 ± 0.8	0.1 ± 0.9	−0.3 ± 0.9	45.2 ± 15.8	1.9 ± 1.7	8.8 ± 0.6	41.5% (37.2–46.0)	1.5 ± 1.7
Colombia (Bogota)	17.6 ± 2.5	−2.3 ± 0.9	−0.5 ± 0.7	−0.1 ± 0.6	68.1 ± 24.8	2.9 ± 1.5	8.8 ± 0.8	23.1% (20.3–26.1)	3.5 ± 2.2
Finland (Helsinki, Espoo, Vantaa)	17.7 ± 2.6	−1.9 ± 0.9	−0.2 ± 0.9	−0.6 ± 0.4	70.7 ± 26.4	2.8 ± 1.7	8.5 ± 0.9	23.8% (20.2–27.7)	3.2 ± 1.7
India (Bangalore)	18.0 ± 3.3	−2.2 ± 1.0	−0.1 ± 0.9	−0.1 ± 0.8	49 ± 21.3	1.8 ± 1.3	8.6 ± 0.7	32.7% (28.8–36.8)	1.8 ± 2.0
Kenya (Nairobi)	17.2 ± 3.1	−2.5 ± 1.1	0.3 ± 1.0	0.2 ± 1.0	71.6 ± 31.3	2.4 ± 1.7	8.6 ± 0.9	19.9% (16.1–23.7)	3.2 ± 2.2
Portugal (Porto)	19.5 ± 3.4	−1.9 ± 0.9	0.2 ± 1.0	−0.4 ± 0.6	56.4 ± 21.6	2.2 ± 1.5	8.3 ± 0.9	46.6% (42.9–50.5)	2.6 ± 1.8
South Africa (Cape Town)	18.0 ± 3.6	−2.3 ± 0.9	0.3 ± 1.1	1.1 ± 1.2	65.1 ± 25.6	3.1 ± 2.1	9.2 ± 0.7	26.9% (23.0–31.2)	3.4 ± 2.4
The United Kingdom (Bath, NE Somerset)	18.5 ± 3.0	−1.7 ± 1.9	0.01 ± 0.9	−0.1 ± 0.7	63.4 ± 22.4	2.9 ± 1.7	9.5 ± 0.7	29.9% (25.8–34.2)	3.2 ± 2.0
The United States (Baton Rouge)	18.9 ± 3.9	−2.3 ± 1.0	−0.1 ± 1.1	0.6 ± 1.3	49.8 ± 18.9	3.1 ± 2.3	8.9 ± 0.9	39.5% (35.2–44.0)	1.8 ± 1.7

BMI = body mass index; MVPA = moderate to vigorous physical activity; PHV = peak height velocity.

**Table 2:** Descriptive statistics (means ± standard deviations, or percentages) for school-level variables stratified by study site.

	Australia (Adelaide)	Brazil (São Paulo)	Canada (Ottawa)	China (Tianjin)	Colombia (Bogota)	Finland (Helsinki, Espoo, Vantaa)	India (Bangalore)	Kenya (Nairobi)	Portugal (Porto)	South Africa (Cape Town)	UK (Bath, NE Somerset)	USA (Baton Rouge)
School Size	541 ± 304	729 ± 595	469 ± 199	1651 ± 717	1625 ± 828	419 ± 133	2378 ± 1609	851 ± 484	820 ± 309	853 ± 288	328 ± 140	790 ± 352
Breaks (<30 min)												
0	7.9%	0.0%	24.4%	0.0%	35.2%	9.3%	0.0%	9.0%	0.0%	4.3%	0.0%	8.1%
1	49.7%	64.0%	9.0%	15.3%	57.1%	0.0%	55.9%	21.3%	8.3%	7.3%	79.7%	50.7%
2	40.7%	22.2%	40.5%	53.6%	7.7%	27.5%	39.4%	54.8%	59.5%	88.5%	20.3%	27.7%
3	1.6%	13.7%	26.1%	31.1%	0.0%	63.2%	4.7%	14.9%	32.2%	0.0%	0.0%	13.4%
Breaks (≥30 min)												
0	5.1%	62.4%	12.2%	0.0%	43.5%	25.9%	0.0%	0.0%	56.3%	87.8%	0.0%	60.3%
1	75.6%	27.9%	35.9%	49.2%	44.1%	60.6%	100%	46.6%	35.6%	5.3%	100%	32.0%
2	17.3%	0.0%	43.7%	0.0%	12.4%	13.5%	0.0%	37.8%	8.2%	6.8%	0.0%	7.7%
3	2.0%	9.7%	8.2%	50.8%	0.0%	0.0%	0.0%	15.5%	0.0%	0.0%	0.0%	0.0%
Promotion of PA												
Yes	100%	95.6%	100%	100%	93.7%	100%	98.4%	91.4%	100%	95.9%	98.1%	100%
No	0.0%	4.4%	0.0%	0.0%	6.3%	0.0%	1.6%	8.6%	0.0%	4.1%	1.9%	0.0%
Promotion of active transportation												
Yes	100%	27.3%	94.1%	68.7%	72.8%	87.9%	67.5%	9.0%	79.2%		96.7%	59.5%
No/don’t know	0.0%	72.7%	5.9%	31.3%	27.2%	12.1%	32.5%	91.0%	20.8%		3.3%	40.5%
PA policies												
Yes	79.8%	94.1%	100%	100%	95.0%	79.2%	100%	93.4%	90.8%	88.0%	95.4%	100%
No	20.2%	5.9%	0.0%	0.0%	5.0%	20.8%	0.0%	6.6%	9.2%	12.0%	4.6%	0.0%
Percentage of students in interschool sports												
Not available/<10%	36.5%	24.6%	6.9%	0.0%	71.1%		22.4%	24.5%	40.8%	1.9%	29.9%	56.2%
10%-24%	43.0%	21.7%	52.3%	32.9%	14.8%		28.4%	10.8%	31.6%	21.4%	29.4%	0.0%
25%-49%	17.7%	24.2%	34.7%	0.0%	7.7%		22.1%	16.1%	12.4%	42.1%	14.8%	16.7%
≥50%	2.9%	29.5%	6.1%	67.1%	6.4%		27.1	48.6%	15.2%	34.6%	25.9%	27.1%
Percentage of students in school or PA clubs												
Not available/<10%	21.8%	26.1%	21.6%	0.0%	60.2%		0.0%	8.8%	8.3%	8.5%	2.7%	45.0%
10%-24%	38.3%	35.6%	27.3%	37.6%	8.6%		6.7%	2.2%	38.2%	19.9%	27.8%	18.3%
25%-49%	17.1%	0.0%	27.5%	32.9%	7.9%		1.6%	13.9%	12.7%	34.0%	47.6%	24.4%
≥50%	22.8%	38.3%	23.7%	29.5%	23.2%		91.7%	75.1%	40.8%	37.6%	21.9%	12.2%
Access to gymnasium (school hours)												
Yes	85.5%	77.4%	100%	64.3%	29.7%	81.0%	39.6%	7.6%	59.9%	13.2%	60.1%	64.2%
No/don’t know	14.5%	22.6%	0.0%	35.7%	70.3%	19.0%	60.4%	92.4%	40.1%	86.8%	39.9%	35.8%
Access playground equipment												
Yes	100%	70.8%	92.4%	30.1%	34.1%	100%	57.1%	75.9%	8.5%	37.4%	91.9%	100%
No/don’t know	0.0%	29.2%	7.6%	69.9%	65.9%	0.0%	42.9%	24.1%	91.5%	62.6%	8.1%	0.0%
Access to gymnasium (outside of school hours)												
Yes	60.3%	18.7%	72.3%	49.0%	14.1%	39.8%	25.1%	17.7%	80.3%	5.3%	34.2%	13.6%
No	39.7%	81.3%	27.7%	51.0%	85.9%	60.2%	74.9%	82.3%	39.7%	94.7%	65.8%	86.4%
Access to indoor facilities (outside of school hours)												
Yes	59.5%	27.6%	53.8%	48.0%	35.6%	25.0%	68.4%	34.5%	48.8%	30.6%	54.0%	13.0%
No	40.5%	72.4%	46.2%	52.0%	65.5%	75.0%	31.6%	65.5%	51.2%	69.4%	45.1%	87.0%
Access to outdoor facilities (outside of school hours)												
Yes	92.3%	2.9%	92.0%	67.7%	50.3%	96.4%	87.3%	71.1%	71.9%	50.2%	72.2%	61.1%
No	7.7%	97.1%	8.0%	32.3%	49.7%	3.6%	12.7%	28.9%	28.1%	49.8%	27.8%	38.9%
Access to equipment (outside of school hours)												
Yes	56.4%	16.1%	25.8%	67.7%	26.7%	21.4%	87.3%	53.6%	53.2%	64.7%	54.1%	30.1%
No	43.6%	83.9%	74.2%	32.3%	73.3%	78.6%	12.7%	46.4%	46.8%	35.3%	45.9%	69.9%

Descriptive information regarding school-level covariates is provided in Table 2. The mean number of students per school ranged from 328 (UK site) to 2378 (India site). In general, differences were noticed in all school variables across the 12 country sites.

**Figure 1:**
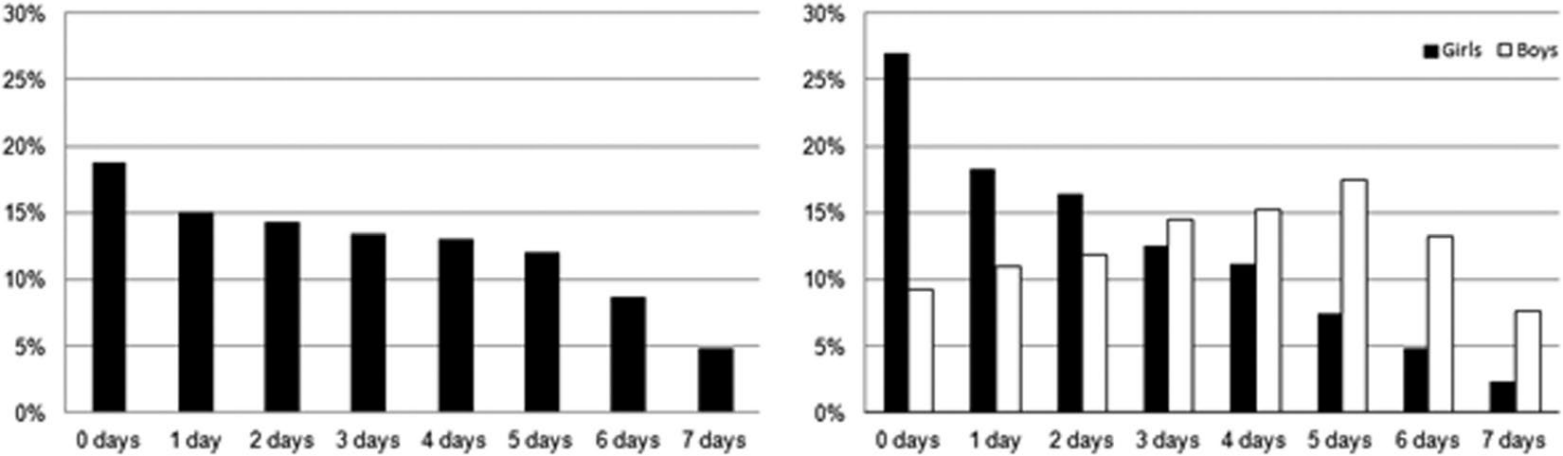
Compliance rates with MVPA recommendations for all children (left panel) and in boys and girls (right panel).

Figure 1 shows compliance rates with MVPA recommendations for all children (left panel) from the 12 country sites. Overall, 4.8% spent ≥60 min in MVPA∙day^−1^ during all 7 days, while 25.5% attained the same level of MPVA on ≥5 days; further, 18.8% did not accumulate ≥60 min in MVPA on any of the monitored days. Sex differences were also evident (right panel). Only 14.6% of the girls complied with the MVPA guidelines for ≥5 days of the week (2.4% complied for all 7 days), and 27% did not comply with the MVPA guidelines on any of the days. Further, boys had higher compliance rates (38.3%) to MVPA guidelines ≥5 days of the week and 7.6% complied all 7 days. The frequency of those boys who did not achieve the daily 60 minutes of MVPA on any of the days (9.2%) was less than that for girls.

Multilevel modelling results are presented in Table 3. Model 1 only included the dummy variables for country treating the Chinese site as the reference. A random child from the Chinese site complied with the recommendations only on 1.54 days∙week^−1^, i.e., roughly 22% of the days, [exp(−1.52) = 0.22 × 7 = 1.54]; children from the United States (US) and Indian sites complied similarly. On the other hand, Portuguese children complied more, at about 3 days∙week^−1^, meaning a rate of 172% [exp(−1.52 + 0.55) = 0.38 × 7 = 2.66] more than their Chinese peers. The country site that complied the most was Finland, where children comply ~4 days∙week^−1^ - a rate of 252% [exp(−1.52 + 0.92) = 0.55 × 7 = 3.85] more than Chinese, followed by the Kenya and Colombia sites (3.56 and 3.64 days∙week^−1^, respectively). Model 2 fit the data significantly better than Model 1 [−2 LogLikelihood (LL) of model 1 = 26711.77 and −2LL of Model 2 = 23396.26; Δ = 3315.51, with 7 df, *P* < 0.0001], and showed that all covariates except healthy diet scores were statistically significant. The final intercept was the estimated percentage of complying days of the Chinese site when all covariates were at zero: briefly, a random Chinese girl complied on 16% of the days, i.e., 1.15 days∙week^−1^. Children from the US site did not differ significantly from the Chinese site. On the other hand, Indian children complied more by a rate of 135.7%, 1.56 days∙week^−1^, while those from the Portuguese site complied ~2 days∙week^−1^. The highest compliance rates were those from the Finnish site (3.11 days∙week^−1^), followed by Australia (2.9 days∙week^−1^), Colombia (2.73 days∙week^−1^), UK (2.63 days∙week^−1^) and Kenya (2.60 days∙week^−1^). On average, boys complied more than girls (rate ratio = 1.47, i.e., 1.47 times greater rate), and overweight/obese children and those closest to their PHV complied less than normal weight ones (rate ratio = 0.81, i.e., a rate reduction of 19%) and their farthest peers (rate ratio = 0.93, meaning a rate reduction of 7%), respectively. Significant positive effects were noticed with unhealthy diet scores (1.08 greater rate), and those accumulating more screen time and with less sleep time had a lower proportion of MVPA compliant days (2% and 4%, respectively).

**Table 3:** Summary of Results of Mixed Models for the number of MVPA complying days.

Parameters	Model 1			Model 2			Model 3		
	β (se)	RR^¥^	95%CI RR^§^	β (se)	RR	95%CI RR	β (se)	RR	95%CI RR
**Fixed effects**									
***Children level***									
Intercept (China site)	−1.52 (0.12)***	0.22	0.17–0.28	−1.77 (0.12)***	0.16	0.13–0.21	−1.80 (0.15)***	0.16	0.12–0.22
USA site	0.21 (0.14)	1.23	0.93–1.62	0.23 (0.13)	1.26	097–1.64	0.38 (0.14)**	1.46	1.10–1.93
India site	0.28 (0.16)	1.33	0.98–1.80	0.31 (0.15)*	1.36	1.02–1.81	0.47 (0.14)**	1.59	1.20–2.12
Portugal site	0.55 (0.14)***	1.72	1.32–2.25	0.60 (0.13)***	1.83	1.43–2.35	0.54 (0.13)***	1.72	1.32–2.23
Brazil site	0.61 (0.14)***	1.83	1.40–2.40	0.68 (0.13)***	1.98	1.54–2.56	0.68 (0.14)***	1.97	1.50–2.60
Canada site	0.65 (0.14)***	1.92	1.47–2.51	0.75 (0.13)***	2.12	1.65–2.72	0.76 (0.14)***	2.15	1.63–2.82
UK site	0.74 (0.14)***	2.10	1.60–2.73	0.83 (0.13)***	2.28	1.77–2.94	0.96 (0.14)***	2.61	1.98–3.46
Finland site	0.92 (0.14)***	2.52	1.93–3.28	1.00 (0.13)***	2.73	2.12–3.50	1.08 (0.14)***	2.95	2.24–3.89
Australia site	0.79 (0.14)***	2.20	1.69–2.87	0.93 (0.13)***	2.55	1.98–3.27	1.05 (0.14)***	2.85	2.16–3.75
Kenia site	0.87 (0.13)***	2.39	1.84–3.10	0.82 (0.13)***	2.28	1.78–2.92	0.93 (0.13)***	2.53	1.96–3.25
South Africa site	0.83 (0.14)***	2.30	1.75–3.01	0.82 (0.13)***	2.26	1.75–2.92	0.93 (0.14)***	2.54	1.93–3.33
Colombia site	0.86 (0.14)***	2.37	1.81–3.09	0.87 (0.13)***	2.39	1.86–3.07	0.85 (0.14)***	2.35	1.79–3.07
Sex				0.38 (0.03)***	1.47	1.38–1.55	0.39 (0.03)***	1.48	1.39–1.57
BMI category				−0.22 (0.02)***	0.81	0.77–0.84	−0.21 (0.02)***	0.81	0.78–0.85
Maturity offset				−0.07 (0.02)***	0.93	0.90–0.96	−0.07 (0.02)***	0.93	0.90–0.96
Unhealthy EP				0.08 (0.01)***	1.08	1.06–1.10	0.08 (0.01)***	1.08	1.06–1.10
Healthy EP				0.01 (0.01)	1.01	1.00–1.03	0.01 (0.01)	1.01	0.99–1.03
Screen time				−0.02 (0.004)**	0.98	0.98–0.99	−0.02 (0.004)***	0.98	0.97–0.99
Sleep				−0.04 (0.01)***	0.96	0.94–0.98	−0.04 (0.01)***	0.96	0.94–0.98
***School level***									
School size							−0.00 (0.00)	1.00	1.00–1.00
Breaks with less than 30 min							0.02 (0.02)	1.02	0.97–1.07
Breaks with 30 or more minutes							0.03 (0.03)	1.03	0.98–1.09
Promotion of students active transportation							−0.02 (0.05)	0.98	0.89–1.08
Access to gymnasium (school hours)							0.04 (0.05)	1.04	0.95–1.14
Access to playground equipment							−0.15 (0.06)***	0.86	0.77–0.96
Access to gymnasium (no school hours)							0.13 (0.05)*	1.14	1.03–1.27
Access to indoor facilities (no school hours)							0.01 (0.05)	1.01	0.92–1.12
Access to outdoor facilities (no school hours)							−0.06 (0.05)	0.94	0.86–1.04
Access to sport equipment (no school hours)							−0.18 (0.05)***	0.83	0.76–0.92
**Random effects**									
Intercept	0.0784 (0.0088)			0.0667 (0.0079)			0.0573 (0.0072)		
−2LogLikelihood	26711.77			23396.26			22186.98		

^¥^Rate Ratios; ^§^95% confidence intervals; ***p < 0.001; **p < 0.01; *p < 0.05.

From the 14 school-variables available in the present study, four of them were excluded in the last modelling step because: (i) the percentage of students involved either in physical activity clubs or in interschool sports was not provided by the Finnish site; (ii) the variability either in school policies or in practices for physical activity across country sites was very low (for example, the percentage of schools with policies or practices for physical activity promotion ranged from 91.4 to 100%). Model 3, with school covariates, fit the data better than Model 2 (−2LL of Model 2 = 23396.26 and −2LL of Model 3 = 22186.98; Δ = 1209.28, with 10 df, *p* < 0.0001). The child-level parameters showed minor changes, and their interpretation remained similar to the previous model. However, at the country level, US children differ from the Chinese, complying more by a rate of 1.46, 1.69 days∙week^−1^. Of the school-level variables included in the model, only three covariates were statistically significant – children’s access to playground equipment, to a gymnasium, and to equipment outside of school hours. On average, children from schools which allowed access to playground equipment or sport equipment had lower MVPA compliance rates of 14% and 17%, whereas children with access to school gymnasium outside of school hours complied more with the daily physical activity guidelines (rate ratio = 1.14, i.e., 14% increased rate).

## Discussion

This study aimed to investigate compliance with MVPA recommendations across a week, as well as to identify its correlates, in a sample of children from a multinational study. The results demonstrated that less than 5% of the total sample complied with the MVPA guidelines every day for a whole week and that approximately one quarter of children attained them on at least 5 days. Cooper *et al*.^5^ investigated daily MVPA compliance in children from ten countries, and the results were quite similar with those observed in the present study, because only 9.0% of the boys and 1.9% of the girls achieved the 60 min∙day^−1^ of MVPA. However, these values are related to the percentage of children achieving the guidelines using only the number of measured/valid days, which ranged from 2 to 7, varying across studies. Further, we could not find the exact number of days children complied with the guidelines during a whole week, apparently because the authors were not able to obtain this information from the studies used. Further, Telford *et al*.^16^ using weekdays and weekend days data from Australian children reported differences in daily MVPA compliance throughout a week, ranging from 16%-39% for boys and 10%-21% for girls. Yet, apparently no information was provided about the precise number of days children complied with the World Health Organization (WHO) recommendation, but only the percentage of children achieving the guidelines on each day of the week. It is of interest to note (in data not shown) that, if we considered mean MVPA of valid/measured days in the current study we would conclude that these children generally complied with the guidelines because their mean value was 60.4 ± 24.8 min∙day^−1^, reinforcing the need to consider each child’s daily results, and not only the average because it does not reflect real day-to-day accumulation patterns.

The results of the multilevel analysis revealed differences in compliance with the MVPA recommendations across countries. Given that the Chinese site was the reference (they had the lowest compliance), children from several other sites complied significantly more. Other multi-country studies^3–5,17^ have also reported differences in children’s MVPA among countries. However, in studies involving only European countries^3,4^, and also data from the International Children’s Accelerometry Database (ICAD)^5^, no clear trend regarding the effect of country-differences in social conditions on children’s physical activity was observed. On the other hand, Onywera *et al*.^17^ reported country-specific data from three nations at different stages of the physical activity transition (Canada, Mexico, Kenya), and Kenyan children had the highest MVPA and that Canadians had the least MVPA. Further, when considering MVPA compliance, although very few studies investigated country differences^3,5^, available results are in line with those considering average MVPA values, suggesting that physical activity levels of children vary across countries. From the most active sites in the present study, three of them are classified as high income countries with high HDI, contrasting with the presumption that children from underdeveloped countries tend to be more physically active^18^. Finnish children were those more likely to comply with the MVPA guidelines and this may be linked to favourable social norms, supportive policies, and high quality walking and cycling infrastructure^11^. Furthermore, children from the most active sites are highly engaged in the use of active transportation to/from school^11^ and this routinized behaviour may help explain their greater daily MVPA compliance. This complex scenario reflects the presence of different clusters of social, cultural, economic and environmental factors acting distinctively in the expression of youth active behaviours, namely their MVPA compliance.

From the set of child-level covariates, girls, overweight/obese children, those closest to their PHV, those with lower unhealthy diet score, and those who accumulate more time in screen time and sleep were less likely to comply with the MVPA guidelines. Sex differences in children’s physical activity levels favouring boys are well-established^2,3,5^, and cultural factors are apparently the main explanation for boys to be involved in more intense activities independent of the country where they live. Although the relationship between physical activity and weight status has been systematically explored and results may be conflicting^19,20^, it is generally accepted that overweight/obese youth tend to spend less time in MVPA, and this trend is observed in different countries^5^. Our data support this general finding, though some disagreement exists about the bi-directional reasoning of this path - some suggest that with increasing adiposity children are less active^19^, while others affirm that children’s lower levels of physical activity tend to increase their adiposity^20^. Regarding biological maturation, a review by Sherar *et al*.^7^ concluded that the relationship (magnitude and signal) between physical activity and biological maturity is inconsistent among studies. However, Malina^21^ highlights that biological maturation can be a factor that affects physical activity during childhood and adolescence, with some studies reporting early mature youth being less active than their later maturing peers, notwithstanding a possible interdependence of other factors, such as BMI, on this relationship^7^.

There may be a positive link between physical activity levels and healthy diets^9^, although available data with youth have mainly focused in the relationship between diet and sedentary behaviours^22^. Reports relating physical activity and diet are apparently contradictory^9,23^. For example, Jago *et al*.^9^ reported that in 10–11-year-old English boys their physical activity was negatively associated with fat intake, but positively associated with total energy and carbohydrate intake; in girls, a positive association was observed between physical activity, fruit and vegetable consumption. On the contrary, Vissers *et al*.^23^ did not find a clear relationship between physical activity and diet, reporting no significant associations in girls, while in boys higher consumption of fizzy drinks and savoury snacks were positively associated with MVPA. In the present study, a positive association between unhealthy diet score and MVPA compliance was observed, meaning that children with higher unhealthy diet scores were more likely to achieve the MVPA guidelines. This result was unexpected; however, the relationship between physical activity and dietary intake is complex, and issues such as dietary compensation may be playing a role in explaining this association^24^.

We found a negative association between screen time and MVPA compliance, and also between sleep duration and MVPA, i.e., children who spend more time in screen activities and who sleep more had a lower rate of MVPA achievement. Although higher amounts of screen time (TV viewing and computer use) has consistently been linked to lower physical activity levels^25^, the same has not been reported regarding sleep. Some studies show higher sleep duration has been related to higher physical activity levels^8^, while others have suggested that more physically active children tend to spend less time sleeping^26^, and that this association varies between countries^26^. One possible explanation for these results is related to the fact that there are only a limited number of hours in the day, such that sleeping longer and spending more time in sedentary activities reduces the available time to be engaged in other activities and, at least, to achieve the MVPA recommendations^27^.

At the school level, only three variables had statistically significant associations with children’s MVPA compliance. Having playground equipment or sport equipment available at the school was negatively related to children’s MVPA compliance; on the other hand, the availability of a school gymnasium outside of school hours was positively associated with it. The results with respect to playground and sports equipment were unexpected, and it is difficult to explain these inverse associations. It could be that the presence of this equipment alone is not as important as having unrestricted access and time available to make use of it during the school day. On the other hand, we speculate that having access to physical activity spaces for unstructured and unsupervised play may be highly beneficial to promote children’s MVPA levels. The school environment can offer students large spaces for physical activities where they may play freely, especially in non-school time, since during school hours they tend to be involved in many seated academic activities.

This study is not without limitations. First, we did not explore children’s physical activity patterns across the whole week, and did not investigate differences in MVPA compliance on school days versus weekend days. Second, although we used an objective instrument to measure physical activity, some physical activities like cycling and swimming were not captured. Third, as per the ISCOLE sampling strategy to maximize variation in socioeconomic status within each site, each sample is not nationally representative. Fourth, the exclusion of children with less than 4 days of valid monitoring may bias the final sample favouring those who complied with the study protocol. Furthermore, in the present study we used ≥4 valid assessment days, and as indicated we included an “offset variable” in our analysis. The offset variable is simply the number of valid assessment days (from 4 to 7) for each observed count. Thus, compliance with MVPA guidelines appropriately considered and controlled for the number of days of measurement. As indicated on data analysis subsection, if two subjects both had two days of MVPA, but their number of valid measurements varied, the statistical model adjusted for this measurement difference appropriately in the statistical analysis. Fifth, the cross-sectional design of the study did not allow for a determination as to whether these relationships change over time. This study also has several strengths, namely its large sample size, with children from countries with different socioeconomic and cultural characteristics comprising the major regions in the world. In addition, computing daily compliance with MVPA recommendations instead of presenting averages of measured days allowed us to better understand children’s daily MVPA patterns. Further, the use of a multilevel analysis with a count model allowed for a better integration of information from diverse origins.

In conclusion, we demonstrated that the daily MVPA guidelines were not achieved by most children in this large multi-national sample of children. Moreover, MVPA compliance varied across the world, namely Chinese, Indian, and US sites’ children had the lowest compliance, and the most compliant were those from Finland, UK and Colombia. Girls, overweight/obese children, those with lower unhealthy diet score, who accumulate more time in sedentariness, and also spend less time sleeping were less likely to comply with the daily MVPA guidelines. Additionally, significant school-level effects were found (but with different signals), namely access to playground equipment, to a gymnasium and to sport equipment outside of school hours. Taken as a whole, these results reflect the important roles country differences, individual characteristics and local school contexts have on children’s daily MVPA accumulation patterns.

## Methods

### Sample

The present sample comes from ISCOLE, a study conducted at sites in 12 countries (Australia, Brazil, Canada, China, Colombia, Finland, India, Kenya, Portugal, South Africa, UK, and US) from all major regions of the world. A detailed account of the study protocol is published elsewhere^28^, as well as participation rates and sampling representation within each country site^29^. It is important to recognize that each ISCOLE data are not representative of each country, but that the sampling strategy was designed to maximize variability in socio-economic status at each site^28^.

A total of 7023 participants (3783 girls; 3240 boys), aged 9–11 years, were sampled. Following the removal of those with missing data, the analytical sample for the current work was 6553. From 60 possible comparisons (12 country sites by 5 variables), of data between included and excluded subjects, by countries, only 12 significant differences were found for the following three variables: maturity offset (Portugal), unhealthy diet (US, Portugal, South Africa, Kenya, China, India), and sleep time (US, Australia, Kenya, Colombia, China). Written informed consent was obtained from parents/legal guardians of all children who were enrolled in the study. The study was in accordance with the Declaration of Helsinki and all procedures were approved by the Pennington Biomedical Research Center (coordinating centre) institutional review board and the local ethics committee(s) of each participating institution. All the methods were performed in accordance with relevant guidelines and regulations.

### Child-level variables

#### Anthropometry

Standing height, sitting height, and weight were measured according to standardized ISCOLE procedures^28^. For standing height and sitting height, children were measured without shoes, with their head positioned in the Frankfurt Plane, using a portable stadiometer (Seca 213, Hamburg, Germany); standing height was measured with children fully erect, feet together, and at the end of a deep inhalation, while sitting height was measured with children seated on a table with legs hanging freely and arms resting on the thighs. Leg length was computed by subtracting sitting height from standing height. Weight was determined using a portable Tanita SC-240 body composition analyzer (Tanita, Arlington Heights, IL, US), with children wearing light clothes and without shoes or socks. Each child was measured twice and, when necessary, a third measurement was taken if the difference between the previous two was outside the permissible range for each measure and its replica (0.5 cm for standing height and sitting height; 0.5 kg for weight). The mean value of closest two measures for each variable was computed and used for analysis.

Body mass index (BMI) was computed using the standard formula [weight(kg)/height(m)^2^], and children were categorized as normal weight or overweight/obese according to cut-points from the WHO based on BMI z-scores^30^.

#### Biological maturation

Biological maturation was computed via maturity offset sex-specific regression equations, which uses age and physical growth characteristics (sitting height, leg length, stature and body mass)^31^. This method estimates, in decimal years, the status of the child relative to their predicted age at peak height velocity (PHV). A positive maturity offset (+) expresses the number of years a child is beyond PHV; a negative maturity offset (−) indicates the number of years before PHV; a zero value indicates that a child is experiencing his/her PHV.

#### Food consumption

Food consumption information was obtained using a Food Frequency Questionnaire (FFQ)^28^, adapted from the Health Behaviour in School-aged Children Survey. For the FFQ, children were asked about the frequency of consumption of 23 different types of food groups in a typical week, with response categories from “never” to “more than once a day”. Based on principal components analysis^32,33^, dietary scores were derived for each child from the children’s FFQ food groups as input variables, expressing children’s dietary patterns. The two components were named “unhealthy” (positive loadings for hamburgers, soft drink, fired food, etc.) and “healthy” (positive loadings for vegetables and fruits, etc.)^32,33^.

#### Physical activity and sleep time

Actigraph GT3X + accelerometers (ActiGraph, Pensacola, FL, US) were used to monitor time spent in MVPA (min·day^−1^) and sleep (hours∙day^−1^). Children were asked to wear the accelerometer at their waist on an elasticized belt placed on the right mid-axillary line 24 hours∙day^−1^, for at least 7 days, including two weekend days. Accelerometer information was divided into daytime activities and nocturnal sleep time using an automated algorithm^34,35^. This algorithm produces more precise estimates of sleep duration, since it captures total sleep time from sleep onset to the end of sleep, including all epochs and wakefulness after onset. Non-wear time during the awake period was defined as any sequence of at least 20 consecutive minutes of zero activity counts^36^. Cut-points advocated by Evenson *et al*.^37^ were used to define time spent in MVPA, which was defined as ≥574 activity counts per 15 second epoch.

To be eligible for primary analyses in ISCOLE^28^, children had to have ≥4 days including ≥1 weekend day (from a whole week, from Monday to Sunday) with a minimum of 10 hours of awake wear time per day. From the 7023 children sampled, a total of 6553 (93.3%) were eligible (3559 girls, 2994 boys). The mean weekly sleep time was determined using valid sleep days (total sleep time ≥160 min) for children with at least 3 valid sleep nights, including at least one weekend night (a total of 6158 children). For the present analysis, our dependent variable was the number of days each child complied with the daily MVPA guidelines during the week; as such, it ranged from 0 (none of the days) to 7 days, and the average of valid days varied from 5.8 (Finnish site) to 6.9 (Chinese site) days.

#### Sedentariness

Sedentariness is a complex characteristic involving behaviours from different domains at work/school, at home, at transportation, and in leisure-time, that include watching TV, using the computer/games, using motorized transportation, and sitting (reading, talking, doing homework, listening to music). In the present study, screen time was used as a proxy measure of sedentariness. This variable was computed based on children’s answers to questions related to time spent watching TV and playing video/computer games or using the computer unrelated to school work, on both school days and weekend^28^. Answers ranged from 1 to 7 (1: 0 hour; 2: less than 1 hour; 3: 1 hour; 3: 2 hours; 5: 3 hours; 6: 4 hours; 7: 5 or more hours). Individual scores were computed such that responses were analogous to number of hours per day (ranging from 0 to 5, with 0.5 representing “less than 1 hour/day”, and 5 representing “5 or more hours per/day). Then, individual scores were weighted and averaged to create mean daily scores ((school day*5 + weekend*2)/7).

### School-level variables

Information concerning the school environment was obtained via a questionnaire (ISCOLE School Environment Questionnaire as presented in Katzmarzyk *et al*.^28^) completed by the physical education teacher or the school principal.

#### School size

School size was defined as the total number of students in each school.

#### School breaks

The number of daily school breaks of less than 30 minutes (category 1) and 30 or more minutes (category 2) was determined. The total number of breaks in each category is described, varying from 0 to 3 + .

#### School policies

Information regarding the existence (yes or no) of school written policies or practices for physical activity promotion was queried; similarly, school promotion of students’ active transportation (allowing children to bring their bicycles) was also binary coded. Percentage of individual students’ involvement in physical activity clubs or interschool sports was also obtained and, in both of them, responses were structured in four categories: not available or <10%; 10–24%; 25–49%; and ≥50%. The categories were dummy coded with “not available or <10%” as the reference category.

#### School facilities and equipment

Information regarding students’ access (yes or no) to school facilities like gymnasia (both during school and outside school hours), indoor and outdoor facilities (outside school hours), and to playground and sport equipment was queried.

### Data analysis

Descriptive statistics are presented as means ± standard deviations (SD) and frequencies. Independent samples t-tests were also used with the purpose to verify putative differences between children included in the present study with those excluded within each country, across the following variables: BMI, maturity offset, healthy and unhealthy diet scores, and mean daily sleep time. These were computed in SPSS 21. Winpepi software was used to compute frequencies and confidence intervals for children’s weight status based on BMI, also for each country.

As the dependent variable is a count (i.e., the number of days a child complied with the recommendations of 60 min of MVPA) a Poisson regression was used^38^. In this model, the count followed a Poisson distribution, and model covariates were related to the log of the expected value of the count. This ensured that the predicted count was positive. Exponentiating the regression coefficients of the Poisson model yields rate ratios, which correspond to ratios of the average count per unit change of the covariate. For example, if the covariate was a dummy for sex (0 = girls, 1 = boys), then the exponentiated regression coefficient represented the average count for boys divided by the average count for girls. Furthermore, given the nature of the clustered data (i.e., children nested within schools) a multilevel Poisson model implemented in SuperMix v.1 software was used^39^. The multilevel model explicitly included a random school effect to account for the effect of the school on the student outcomes, i.e., the use of multilevel models allowed modelling the relationship between children’s MVPA, their individual characteristics and school environmental factors. Thus, students within the same school were allowed to be correlated, and the magnitude of the (variance of the) random school effect indicated the degree of correlation of the students within schools. Given that children from all sites did not have the same number of valid days of measurement, an offset term to adjust for these differences in number of valid days (from 4 to 7) was included in all models^38^. Thus, if two subjects each had two days of MVPA, but their number of valid measurement days differed (say four and seven days), the offset (i.e., number of valid measurement days) ensured that the MVPA rates differed for these two subjects (2/4 vs 2/7). Based on an iterative procedure, all parameters (fixed and random) were simultaneously estimated using a full maximum likelihood approach as described elsewhere^40^. Individual (level-1) covariates included sex (girls were the reference category), BMI (reference was normal weight), maturity offset (as a continuous variable), diet, screen time, and sleep; school (level-2) predictors included school size, number of school breaks (with less than 30 minutes, and with 30 or more minutes), school promotion of students’ active transportation, and students’ access to (1) gymnasium during school hours and outside of school hours, (2) playground equipment, (3) indoor and outdoor facilities outside of school hours, and (4) sport equipment (for all these variables, the answer “yes” was used as the reference category). Finally, because of the relatively small number of sites, dummy variables for country sites were included to account for differences across the twelve country sites (rather than treating country site as a third level in the multilevel model) as advocated by Snijders and Bosker^41^. The statistical modelling was based on a threefold approach: step 1 (model 1) only included the dummy variables for country sites; in step 2 (model 2), children’s characteristics were added as covariates to identify possible modifications in the parameter estimates of model 1; in step 3 (model 3), school (level-2) predictors were added. Significance level was set at 5%.

### Data availability

Due to the ethical restrictions placed by the Ethics Committees from the 12 countries, as well as data proprietorship by the coordinating centre at Pennington Biomedical Research Center in Baton Rouge, data cannot be made publicly available. Data will be available upon request, and individuals or readers interest in the data can contact ISCOLE coordinating center (Peter.Katzmarzyk@pbrc.edu).
